# Effectiveness and safety of gonadotropins used in female infertility: a population-based study in the Lazio region, Italy

**DOI:** 10.1007/s00228-022-03330-1

**Published:** 2022-05-04

**Authors:** Alessandro Cesare Rosa, Arianna Pacchiarotti, Antonio Addis, Andrea Ciardulli, Valeria Belleudi, Marina Davoli, Ursula Kirchmayer

**Affiliations:** 1 Department of Epidemiology, Regional Health Service, ASL Roma 1, Rome, Lazio Italy; 2 Medically Assisted Procreation Center, Ospedale San Filippo Neri, Rome, Italy; 3 Department of Obstetrics and Gynecology, Division of Maternal and Fetal Medicine, Ospedale Cristo Re, Rome, Italy

**Keywords:** Gonadotropins, Infertility, Administrative health data, Real-world evidence, Cohort, Effectiveness, Safety

## Abstract

**Purpose:**

Infertility is a topic of growing interest, and female infertility is often treated with gonadotropins. Evidence regarding comparative safety and efficacy of different gonadotropin formulations is available from clinical studies, while real-world data are missing. The present study aims to investigate effectiveness and safety of treatment with different gonadotropin formulations in women undergoing medically assisted procreation treatments in Latium, a region in central Italy, through a real-world data approach.

**Methods:**

A retrospective population-based cohort study in women between the ages of 18 and 45 years who were prescribed with at least one gonadotropin between 2007 and 2019 was conducted. Women were enrolled from the regional drug dispense registry, and data on their clinical history, exposure to therapeutic cycles (based on recombinant “REC” or extractives “EXT” gonadotropin, or combined protocol “CMD” (REC + EXT)), and maternal/infantile outcomes were linked from the regional healthcare administrative databases. Multivariate logistic regression models were applied to estimate the association between exposure and outcomes.

**Results:**

Overall, 90,292 therapeutic cycles prescribed to 35,899 women were linked to pregnancies. Overall, 15.8% of cycles successfully led to pregnancy. Compared to extractives, recombinant and combined treatments showed a stronger association with conception rate (RR_REC_ adj = 1.06, 95% CI: 1.01–1.12; RR_CBD_ adj = 1.17, 95% CI: 1.11–1.24). Maternal outcomes occurred in less than 5% of deliveries, and no significant differences between treatments were observed (REC vs EXT, pre-eclampsia: RR adj = 1.24, 95% CI: 0.86–1.79, ovarian hyperstimulation syndrome: RR adj = 1.25, 95% CI: 0.59–2.65, gestational diabetes: RR adj = 1.06, 95% CI: 0.84–1.35). Regarding infantile outcomes, similar results were obtained for different gonadotropin formulations (REC vs EXT: low birth weight: RR adj = 0.98, 95% CI: 0.83–1.26, multiple births: RR adj = 1.06, 95% CI: 0.92–1.23, preterm birth: RR adj = 1.03, 95% CI: 0.92–1.26).

**Conclusions:**

Efficacy and safety profiles of REC proved to be similar to those of EXT. Regarding the efficacy in terms of conception rate and birth rate, protocols using the combined approach performed slightly better. Outcomes related to maternal and infantile safety were generally very rare, and safety features were overlapping between gonadotropin formulations.

## Background

According to the World Health Organisation, infertility affects up to 15% of reproductive-aged couples worldwide [1], and the same percentage has been estimated also in Italy [2]. Among couples hit by infertility, in most cases, causes can be attributable to either males (qualitative and quantitative alterations of semen parameters) or females (tubal disease, ovulatory disorders, endometriosis), while in approximately 15% of couples’ infertility remains unexplained Carson and Kallen [3].

The assisted reproductive technology (ART) was born in order to help couples with infertility issues in having a baby. The first treatments of in vitro fertilisation used the spontaneous cycle of the women, with the retrieval of only one oocyte. Further studies have shown that it is possible to induce ovulation by administrating gonadotropins (Gonas) during the menstrual cycle, in order to obtain a higher number of oocytes.

The availability of different Gona formulations in terms of extractive (highly purified) urinary Gonas (EXT) and recombinant Gonas (REC), and the authorisation of follitropin alpha biosimilars make the choice of the individual treatment complex.

Evidence from clinical studies regarding different efficacy and safety profiles of urinary versus recombinant formulations is controversial: some authors report findings which favour one formulation over the other: a paper by Out et al. [4] states favourable results in terms of pregnancy among women treated with recombinant FSH. And there is also evidence for advantages in using a combined protocol of both formulations (CBD): a prospective randomised study Pacchiarotti et al. [5] showed that using a combination of both urinary and recombinant FSH for ovarian stimulation improves oocyte maturity and embryo cleavage and increases pregnancy and implantation rates. The advantage of the combined protocol over the single formulations was confirmed by a review of literature Pacchiarotti et al. [6]. At the same time, the risk of ovarian hyperstimulation syndrome was observed to be more frequent in women treated with recombinant formulations with respect to extractives Pachiarotti et al. [7].

A recent review of the results of comparative clinical trials, Cochrane analyses, and meta-analyses concluded that the available evidence from the published literature does not show significant differences between urinary and recombinant gonadotropins in terms of safety and efficacy Patki et al. [8]. In the absence of differences in efficacy and safety, several authors recommend considering other factors when choosing a gonadotropin regimen, such as costs, patient acceptability, and drug availability [9–11].

Since their introduction, recombinant Gonas have steadily gained market, outranging the use of urinary formulations. While in the first years, this might have been partly explained by better patient acceptability due to a simpler administration (subcutaneous) with respect to the urinary formulations (intramuscular), and this does not hold true in recent years, as almost all urinary formulations are administered subcutaneously now.

Initial disadvantages of recombinant formulations due to higher costs with respect to extractives have been mainly overcome after the introduction of the recombinant FSH biosimilar, which led to price reductions of the originator as well.

To date, evidence from real-world settings is missing, but might add useful information for clinicians, when choosing the treatment protocol for their patients, and for policy makers, when deciding on recommendations and targeting policies with respect to refunding the different formulations.

The present study tries to close the gap on evidence under real-world conditions and provide evidence through an observational population-based approach, aiming to investigate the use patterns of the different Gona formulations and perform a comparative evaluation of the risk–benefit profile of different formulations of Gonas used for ovarian stimulation in female infertility, to answer to the question if recombinant formulations present advantages over urinary formulations.

## Methods

### Study design

We performed a retrospective observational population-based study.

### Data sources

The present study used data from the administrative healthcare databases of the Lazio region, namely: the regional healthcare assistance file, which contains demographic and residence information about all residents alive and registered in the regional health service at a specific year’s date. The regional drug dispensing registry (Pharm). The registry is limited to drugs dispensed to outpatients and reimbursed by the healthcare system. Drugs are identified by the national drug register code, which refers to the international Anatomical Therapeutic Chemical Classification System (ATC). Individual patient data and the date of drug claim are reported for every prescription. The hospital information system (HIS), which provides, for every hospitalisation in a regional hospital, information on patients’ demographic characteristics, discharge diagnoses, and procedure codes according to the International Classification of Disease, Ninth Revision, Clinical Modification (ICD-9-CM). The regional mortality information system (MIS), providing information on demographic characteristics, as well as date, place, and cause of death (codified by ICD-9 codes). The regional database of birth certificates [12], which collects information of socio-demographic (mother) and health (newborn) status referring to all deliveries in the region.

### Study population

From administrative healthcare databases, all drug claims of Gonas to women enrolled in the regional healthcare system in the 24 months before each drug claim (characterisation period), and aged 18–45 years were retrieved for the period 2007–2019. Women treated with human chorionic gonadotropin (HCG) only or with more than 9 treatment cycles were excluded. Our unit of observation was the treatment cycle, which was defined as a 21-day mobile time window (Fig. 1). Thus, each woman could contribute with up to 8 observations. The analysis related to the outcomes comes as far as the updating of the information source, CeDAP, permits (births occurring through 2018).

**Fig.**
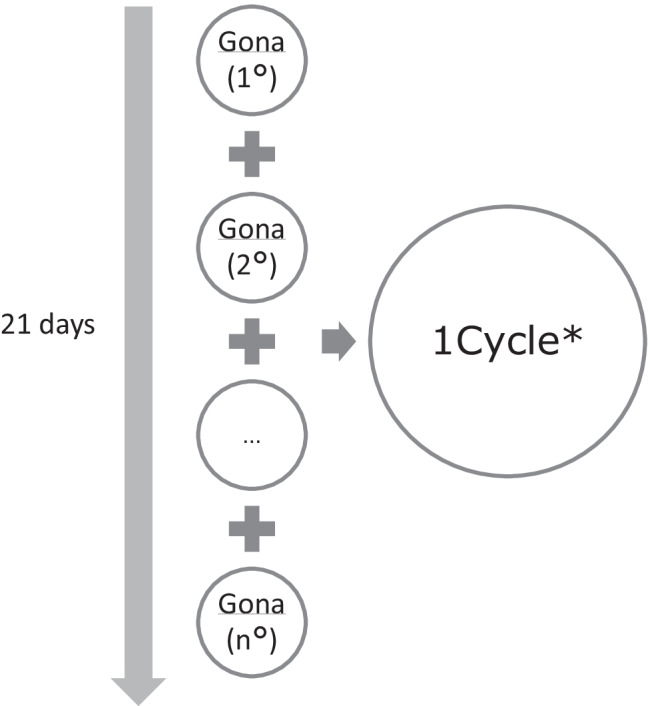
1 Definition of the treatment cycle from drug dispensing data *For each assisted woman in the Lazio region, therapeutic cycles are identified based on the dates of drug delivery in a 21-day mobile window. The drugs assigned to each window allow to associate one of the following formulations to each cycle: recombinant, extractive, combined (recombinant + extractive)

### Exposure

All Gonas dispensed in the study period were retrieved, considering the ATC group G03GA (Appendix Table 3). Exposure was defined based on the treatment cycle, i.e., the 21-day mobile time window and classified into treatment with EXT, REC, or a combination of both (CBD) (Fig. 1).

### Validation of the treatment cycle

The availability of the data gathered in the Medically Assisted Procreation Center of the San Filippo Neri Hospital in Rome (in accordance with the GDPR procedure), referring to patients treated between 2016 and 2019, allowed to evaluate the concordance between cycles registered in the clinical dataset and those retrieved from administrative data, for the same woman, in particular in relation to the definition of the therapeutic cycle (REC, EXT, CBD).

### Follow-up

For each treatment cycle, the observation started on the day of the first drug dispensing and ended at the first occurrence of study outcome, death, or loss to follow-up.

### Outcomes

#### Effectiveness

From HIS and CeDAP, pregnancies, both ending in abortion and in childbirth, were retrieved and linked with cycles within the 9 months from the last Gona claim.

#### Safety outcomes

##### Infantile

From CeDAP, the following parameters were considered: number of births, caesarean birth, twin births, sex ratio, preterm births, weight/length/cranial circumference at birth, Apgar score, still birth, and admission to emergency neonatal therapy (Appendix Table 5).

##### Maternal

From HIS pre/eclampsia, ovarian hyperstimulation syndrome and gestational diabetes were retrieved (Appendix Table 4).

### Confounding

A pre-defined list of potential confounders (based on the literature Bateman et al. [13] and integrated in collaboration with clinicians) and measures of overall status of women and socio-demographic characterises, at the moment of each therapeutic cycle, was taken into account for risk-adjustment when looking at their healthcare outcomes (Appendix Table 4).

### Analysis

#### Treatment patterns

For each woman, the transition between treatment cycles from the first up to max the fourth cycle was described using a Sankey diagram. After the first cycle, classified as one of the three exposure categories, the second event could be either pregnancy (abortion or birth), a second cycle (categorised in the three exposure categories), or the end of treatment. The same was investigated for those women undergoing a second treatment cycle, and so on.

#### Efficacy and safety

The association between exposure to EXT, REC, CBD, and each outcome was investigated applying log-binomial regression models.

## Results

**Fig. 2:**
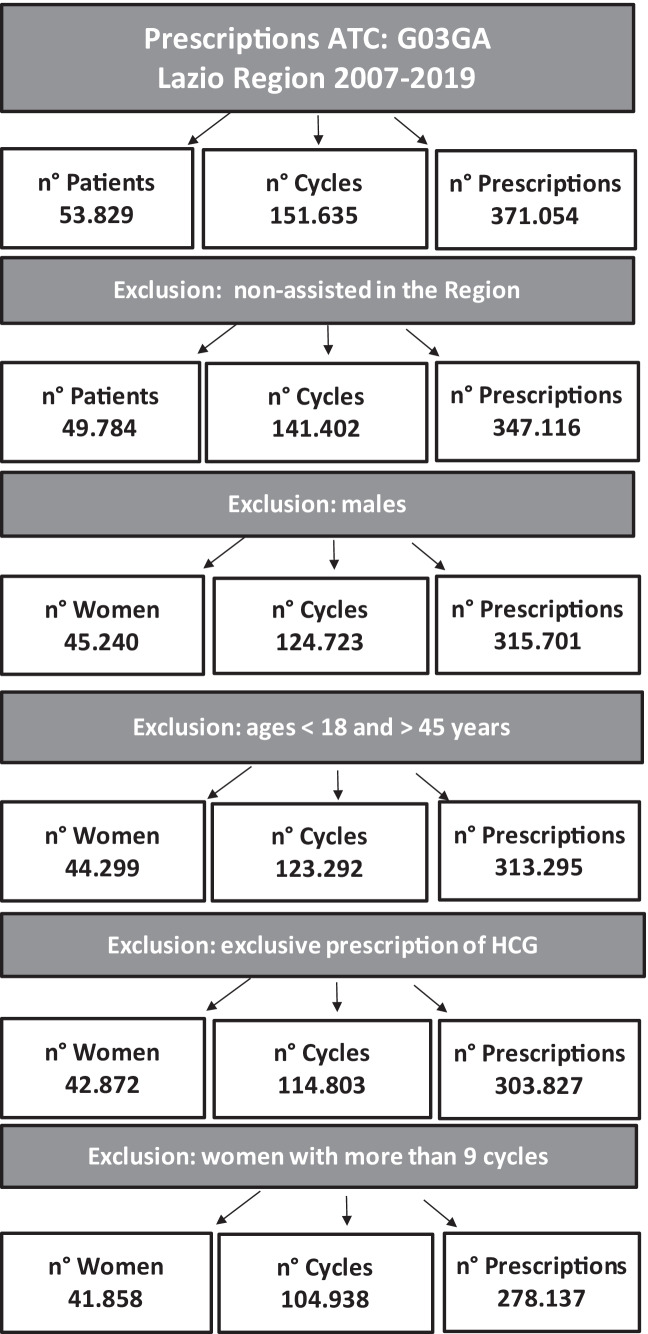
Flow-chart of the study cohort: women, cycles, and drug dispensing

After applying inclusion and exclusion criteria, 104,938 cycles prescribed to 41,858 women were retrieved (Fig. 2). The validation performed through linkage with 268 clinical records shows satisfactory concordance with our approximation (*K* = 0.9017). On average, women underwent 2.51 cycles and each cycle was defined based on 2.65 prescriptions. Accounting for the availability of data from birth certificate up to the end of 2018, and allowing 12 months of observation, our cohort included a total of 90,292 cycles related to 35,899 women (Table 1). Of these, almost half were REC, and a quarter EXT or CBD, respectively. Analysing the characteristics of women prescribed with different cycles, we found that women over 40 were prescribed more often with CBD (37.9%) and EXT cycles (34.0%) compared to REC (30.9%). In terms of access to healthcare, we observed differences in use of co-medication and access to ambulatory visits during the 6 months preceding the cycles, with REC cycles attributable to lower proportions. In general, women included were in good health condition. The only comorbidities with higher frequency are those which can be considered related to resorting to ART—like female infertility, cervical polyp, and endometriosis (Table 2).

**Table 1:** Characteristics of the study cohort

	**Gonadotropin cycles (2007–2017)**							
	**REC**		**EXT**		**CBD**		**Total**	
	***N* = 45,554 (50.5%)**		***N* = 24,509 (27.1%)**		***N* = 20,229 (22.4%)**		***N* = 90,292**	
**Age of woman**		**%**		**%**		**%**		**%**
18–24	303	0.7	136	0.6	50	0.2	489	0.5
25–29	2731	6.0	1146	4.7	750	3.7	4627	5.1
30–34	10,608	23.3	5079	20.7	3654	18.1	19,341	21.4
35–39	17,825	39.1	9817	40.1	8115	40.1	35,757	39.6
40–45	14,087	30.9	8331	34.0	7660	37.9	30,078	33.3
**Measures of overall health status (6 months prior the index date)**								
*Number of co-medication (distinct ATC at 4 level)*								
0	6954	15.3	4166	17.0	2155	10.7	13,275	14.7
1–3	17,339	38.1	9799	40.0	6414	31.7	33,552	37.2
4–7	12,737	28.0	6507	26.5	5844	28.9	25,088	27.8
8 +	8524	18.7	4037	16.5	5816	28.8	18,377	20.4
*Prior hospitalisation*								
Yes	7141	15.7	3406	13.9	2978	14.7	13,525	15.0
*Prior emergency room visits*								
Yes	4524	9.9	2392	9.8	2059	10.2	8975	9.9
*Number of prior outpatient visits*								
0	22,158	48.6	9232	37.7	9609	47.5	40,999	45.4
1–2	12,006	26.4	6457	26.3	5595	27.7	24,058	26.6
3–8	9719	21.3	7229	29.5	4273	21.1	21,221	23.5
9 +	1671	3.7	1591	6.5	752	3.7	4014	4.4
**Comorbidities**								
Alcohol abuse	19	0.0	3	0.0	9	0.0	31	0.0
Asthma	51	0.1	29	0.1	23	0.1	103	0.1
Cardiac valvular disease	19	0.0	9	0.0	5	0.0	33	0.0
Gestational diabetes mellitus	244	0.5	114	0.5	104	0.5	462	0.5
Preexisting diabetes mellitus	54	0.1	34	0.1	26	0.1	114	0.1
Drug abuse	11	0.0	5	0.0	5	0.0	21	0.0
Severe pre-eclampsia/eclampsia	39	0.1	20	0.1	14	0.1	73	0.1
Mild or unspecified pre-eclampsia	83	0.2	28	0.1	42	0.2	153	0.2
Endometriosis	1243	2.7	566	2.3	541	2.7	2350	2.6
Polycystic ovary syndrome	52	0.1	35	0.1	15	0.1	102	0.1
Sickle cell disease	20	0.0	32	0.1	13	0.1	65	0.1
Cystic fibrosis	5	0.0	5	0.0	2	0.0	12	0.0
HIV	15	0.0	16	0.1	11	0.1	42	0.0
Female infertility	8309	18.2	3555	14.5	3432	17.0	15,296	16.9
Chronic ischemic heart disease	0	0.0	0	0.0	0	0.0	0	0.0
Pulmonary hypertension	0	0.0	0	0.0	0	0.0	0	0.0
Preexisting hypertension	204	0.4	109	0.4	110	0.5	423	0.5
Gestational hypertension	166	0.4	72	0.3	89	0.4	327	0.4
Chronic ischemic heart disease	8	0.0	7	0.0	6	0.0	21	0.0
Systematic lupus erythematosus	11	0.0	4	0.0	4	0.0	19	0.0
Congenital heart disease	22	0.0	13	0.1	12	0.1	47	0.1
Chronic renal disease	37	0.1	12	0.0	14	0.1	63	0.1
Multiple gestation	1019	2.2	420	1.7	450	2.2	1889	2.1
Obesity	100	0.2	49	0.2	33	0.2	182	0.2
Placenta previa	141	0.3	68	0.3	65	0.3	274	0.3
Cervical polyp	2934	6.4	1640	6.7	1417	7.0	5991	6.6
Previous caesarean delivery	40	0.1	27	0.1	14	0.1	81	0.1
Tobacco use	2	0.0	1	0.0	0	0.0	3	0.0
Tumours	167	0.4	84	0.3	67	0.3	318	0.4

**Fig. 3:**
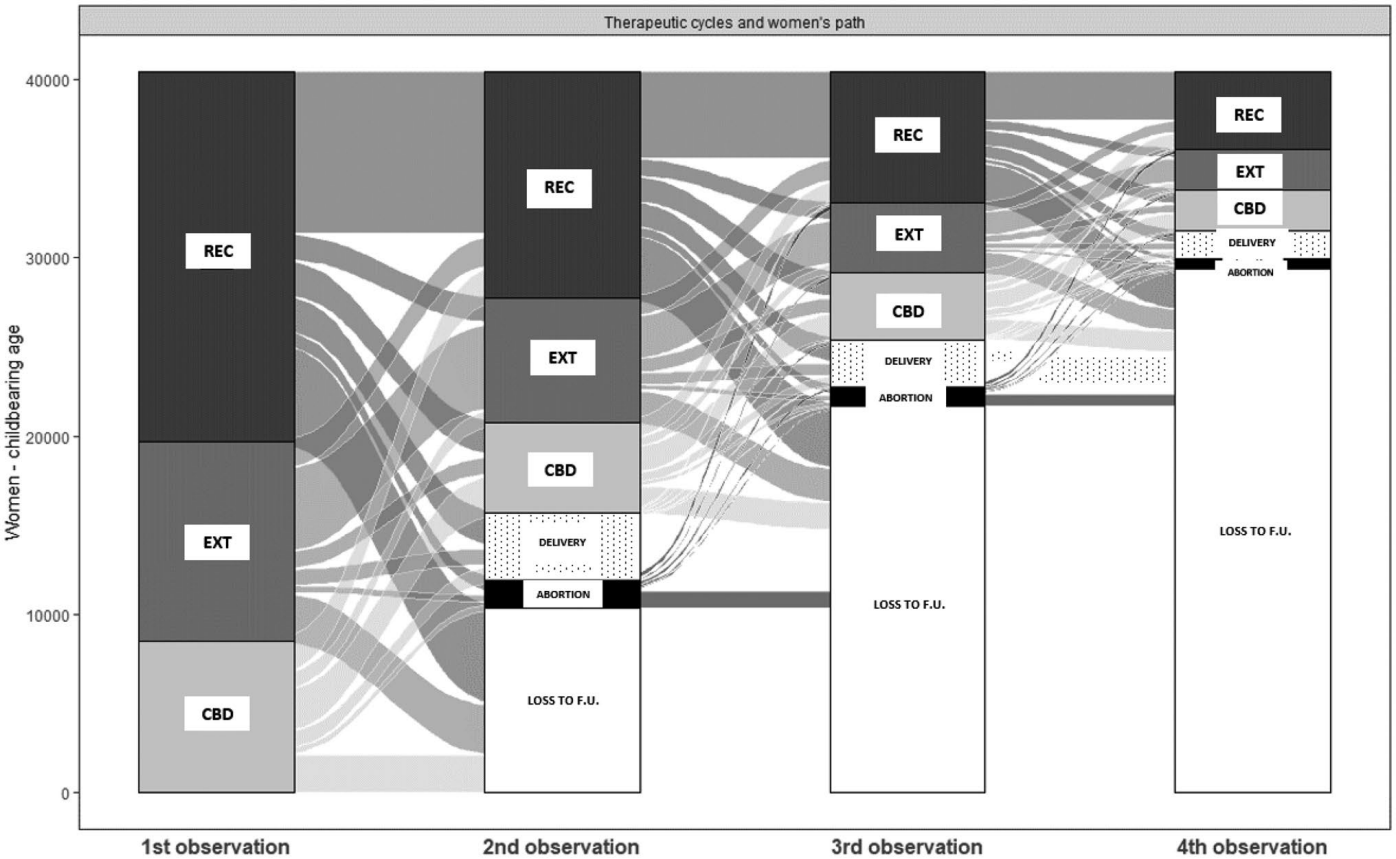
Pathway analysis: treatment patterns and transition between treatments and pregnancy (abortion and pregnancy)

Through the alluvial diagram, it is possible to map the main events of a woman within a path of ART and visualise the attempts she makes to obtain a pregnancy. The magnitude of each stream represents the proportion of women moving from a status to another (Fig. 3).

Each woman is observed over time. The 1st observation represents the proportion of women starting with the first gonadotropin-based drug cycle: 51.5% REC, 26.3% EXT, 22.2% CBD.

In subsequent observations, it is possible to verify whether a woman obtains the desired result (child delivery, box filled with points), reaches pregnancy followed by abortion (black category), or continues the treatment with further attempts (same therapy or switch). In the white category, “loss to follow-up,” we observe women for whom we have no more information. The same setting is repeated for subsequent observations.

The 2nd observation shows that 15.2% of women successfully achieve a pregnancy at first attempt (whether it results in a delivery or an abortion), with differences between Gona types, i.e., 15.9% for women exposed to REC, 12.7% to EXT, and 17.0% to CBD.

Considering the entire pathway cumulatively, even beyond the 4th event, 28.4% of women overall achieve a pregnancy with delivery (this accounts for 11.6% of therapeutic cycles).

23.3% of women are lost to follow-up after the 1st observation. Most women who achieve pregnancy over time do not encounter additional information from information systems.

Table 2 presents the frequencies of treatment success and maternal and infantile outcomes for the three exposure categories.

**Table 2:** Cycles related to pregnancy, characteristics of outcomes

	**Gonadotropin cycles (2007–2017)**							
	**REC**		**EXT**		**CBD**		**Total**	
	***N* = 45,554 (50.5%)**		***N* = 24,509 (27.1%)**		***N* = 20,229 (22.4%)**		***N* = 90,292**	
***Effectiveness outcomes***								
		**%**		**%**		**%**		**%**
**Pregnancy interrupted**	1907	4.2	970	4.0	902	4.5	3779	4.2
**Pregnancy (birth rate)**	5475	12.0	2556	10.4	2472	12.2	10,503	11.6
**Conception rate**	7382	16.2	3526	14.4	3374	16.7	14,282	15.8
Number of cycles to get to a pregnancy/pregnancy interrupted								
1	3232	43.8	1421	40.3	1415	41.9	6068	42.5
2	2023	27.4	1037	29.4	788	23.4	3848	26.9
3	1002	13.6	492	14.0	526	15.6	2020	14.1
4 +	1125	15.2	576	16.3	645	19.1	2346	16.4
Safety outcomes* (child)								
**# of children at delivery**								
1	4452	81.3	2131	83.4	2014	81.5	8597	81.9
**Multiple births**	1023	18.7	425	16.6	458	18.5	1906	18.1
2	958	17.5	394	15.4	437	17.7	1789	17.0
3 +	65	1.2	31	1.2	21	0.8	117	1.1
**Caesarean section**	3540	64.7	1615	63.2	1559	63.1	6714	63.9
**Born alive**	5465	99.8	2552	99.8	2461	99.6	10,478	99.8
**Sex**								
Male	3069	51.6	1395	50.3	1387	51.4	5851	51.2
Female	2877	48.4	1378	49.7	1314	48.6	5569	48.7
Not available	2	0.0	2	0.1	0	0.0	4	0.0
**Small for gestational age (SGA)**	900	16.4	450	17.6	411	16.6	1761	16.8
**Low birth weight (LBW)**	1282	23.4	568	22.2	559	22.6	2409	22.9
**Preterm**	1222	22.3	539	21.1	542	21.9	2303	21.9
**Apgar score:**								
* Excellent condition*	5291	96.6	2499	97.8	2407	97.4	10,197	97.1
* Moderately depressed*	65	1.2	22	0.9	20	0.8	107	1.0
* Severely depressed*	118	2.2	35	1.4	44	1.8	197	1.9
* Apgar missing*	1	0.0	0	0.0	1	0.0	2	0.0
**Admission to emergency neonatal therapy**	601	11.0	274	10.7	281	11.4	1156	11.0
Safety outcomes* (mother)								
**Ovarian hyperstimulation syndrome (OHSS)**	35	0.6	14	0.5	15	0.6	64	0.6
**Pre-eclampsia**	118	2.2	43	1.7	53	2.1	214	2.0
**Gestational diabetes**	228	4.2	105	4.1	96	3.9	429	4.1

*Denominator refers to cycles related to births

The conception rate, i.e., successful achievement of pregnancy, independently from childbirth, was highest for CBD cycles (16.7%) and lowest for EXT (14.4%). On the contrary, the number of cycles needed to achieve pregnancy was higher in the CBD group (19.1%).

Regarding the effectiveness of therapeutic treatment, REC cycles, compared to EXT, showed a higher association with pregnancy (conception rate RR adj 1.06, 95% CI: 1.01–1.12) and with childbirth (birth rate, RR adj 1.07, 95% CI: 1.02–1.13). The association was stronger when we compared the effectiveness of CBD vs EXT cycles: conception rate RR adj 1.17, 95% CI: 1.11–1.24 and birth rate, RR adj 1.19, 95% CI: 1.12–1.27.

The characteristics of deliveries related to treatment with different Gona formulations were similar. Generally, multiple births are considerably more frequent in medically assisted pregnancies compared to the overall population, independently of the type of Gona used (18.1% respect to 1.8% in the Lazio birth register (13CeDAP)). Caesarean section was applied in almost 64% of deliveries, about one-fifth of children were born at preterm, and 11% were treated in emergency neonatal therapy. We observed very few stillbirths (0.2%), and most children were in excellent health conditions at birth (97.1%). The three maternal outcomes considered, occurred rarely (4.1% gestational diabetes, 2.0% pre-eclampsia, and 0.6% ovarian hyperstimulation syndrome), with similar proportions across exposure categories.

**Fig. 4:**
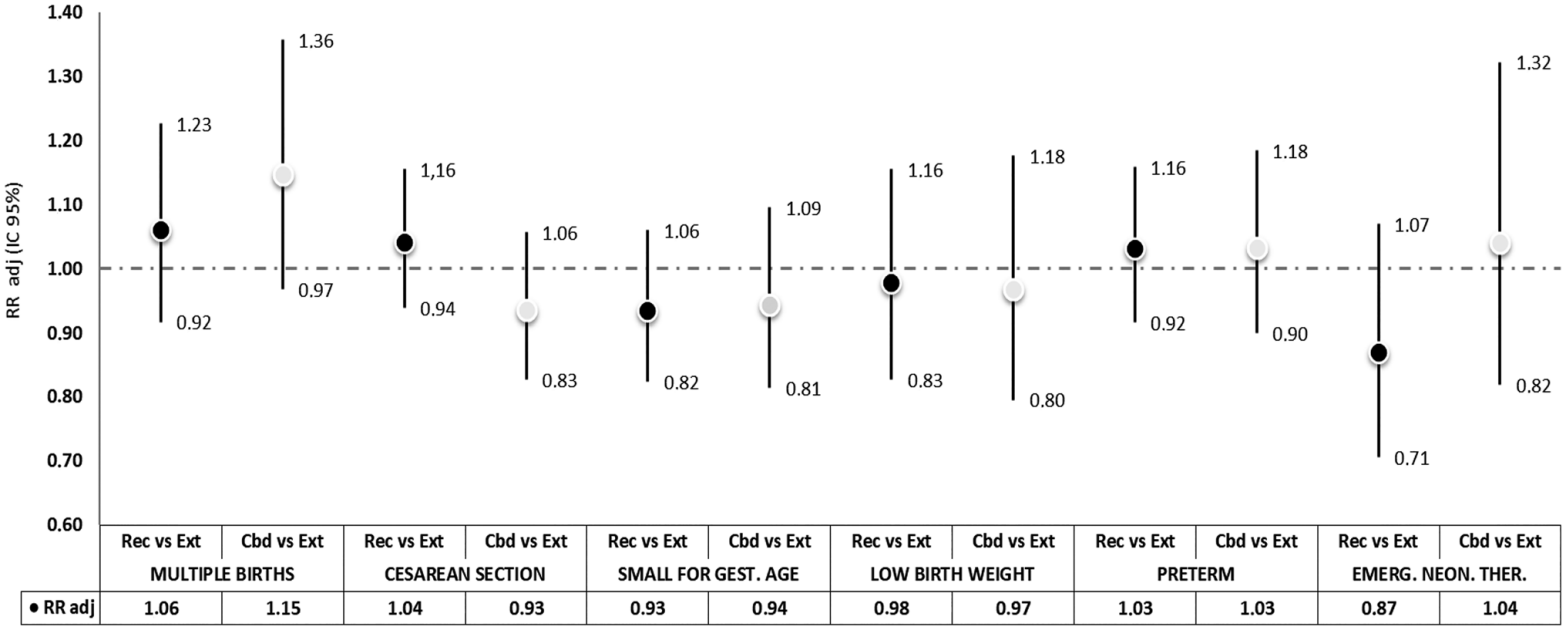
Association between exposure to the three exposure categories and infantile outcomes

**Fig. 5:**
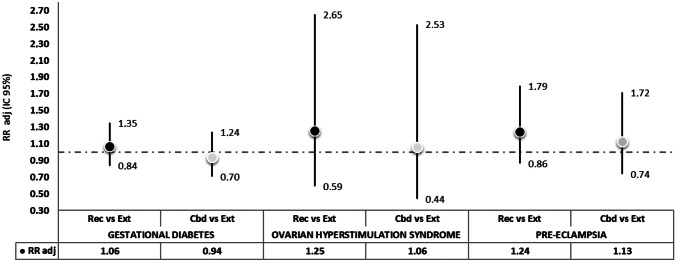
Association between exposure to the three exposure categories and infantile outcomes

Results of the regression models investigating maternal and infantile outcomes are presented in Figs. 4 and 5, respectively, comparing REC and CBD treatments to EXT treatment. Regarding infantile outcomes, similar results were obtained, and all estimates included the null difference level. Yet, some slight advantages were found for REC and CBD treatments: for both with respect to relative and absolute measures of neonatal weight and length (SGA: RR_REC_: 0.93, RR_CBD_: 0.94), for CBD treatments regarding caesarean section (RR_CBD_: 0.93), and for REC treatment with respect to admission to neonatal emergency therapy (RR_REC_: 0.87). For all maternal outcomes, point estimates were slightly higher for REC and CBD cycles, but no significant differences were observed. At the same time, CBD and REC cycles were associated with a slightly increased risk for preterm (RR_REC_/_CBD_: 1.03) and multiple births (RR_REC_: 1.06, RR_CBD_: 1.15).

## Discussion

In the present study, efficacy and safety profiles of recombinant gonadotropins have proved to be like those of extractive gonadotropins. Regarding the efficacy in terms of conception rate and birth rate, protocols using the combination of both formulations were found to perform slightly better. Outcomes related to maternal and infantile safety were generally very rare, and safety features were very similar between Gona formulations.

Our findings of overlapping safety and efficacy are in line with the available evidence provided by clinical studies, reviews, and meta-analyses. The higher efficacy of the mixed approach with respect to monotherapy confirms findings reported in the Italian study Pacchiarotti et al. [5] based on clinical data.

As contextual information, the average number of therapeutic cycles per woman is in line with the refund policies in use in the Lazio region.

The added value of our study stems from its nature, being, to our knowledge, the first observational approach performed in a real-world setting and comprising treatments offered at the level of the resident population over a long period, granting a robust number of observations. This was possible thanks to the availability of administrative healthcare data and the collaboration in a multidisciplinary approach, comprising epidemiologists, statisticians, and specialist clinicians.

Still there are some limitations to be mentioned. Most importantly, our data do not comprise information on treatment cycles, and consequently, we had to develop an algorithm for its approximation. Fortunately, we had the opportunity to use data from a clinical setting which allowed to evaluate the performance of our algorithm, and this comparison proofed satisfactory concordance about the categorisation of the cycle.

Another weakness of our data stems from the lack of information regarding clinical details, and lifestyle data. This may have multiple impacts: first, it limits the choice of the outcomes we can measure. For example, our information on spontaneous abortions is restricted to cases requiring access to emergency room or hospital. This probably implies an underestimation of the conception rate. Similarly, intermediate treatment results, such as oocyte maturity or embryo cleavage, cannot be considered. Second, outcomes may be sensitive to different doses of gonadotropins, an information which is not currently available from our administrative data. Third, we do not have the possibility to distinguish different levels of medically assisted procreation with the right accuracy level. Last, we cannot account for some potential confounders such as BMI, alcohol consumption, or smoking. Yet, we do not expect any differences in the prevalence of these latter variables between treatment regimens.

To date, there are no agreed treatment guidelines which favour one gonadotropin formulation over the other, enhance combined treatment, or recommend specific doses. Treatment choices are rather made by the specialist physician upon a complex evaluation of the specific case, which accounts not only for clinical aspects of the woman to be treated but also for parameters referring to the partner. These considerations cannot be retrieved in our data and leave space for indication bias.

Taken all these considerations together, we believe that the findings of our study add an important piece of information to the complex field of infertility treatment with gonadotropins, both for clinicians and health policy makers.

Future research might investigate the cost–benefit profiles of the different Gona formulations, to complete the picture.

## Data Availability

The data analysed in the current study are not publicly available due to privacy issues but are available from the corresponding author on reasonable request.

## Appendix

**Table 3:** Gonadotropins (G03GA): list of active agents

Gonadotropins	ATC	Active substance	Originator	Biosimilar
uHCG	G03GA01	**Chorionic gonadotropin**	Gonasi	
			Pregnyl	
uHP-hMG	G03GA02	**Human menopausal gonadotropin (menotropin)**	Meropur	
			Meriofert	
uFSH	G03GA04	**Urofollitropin**	Fostimon	
rFSH	G03GA05	**Follitropin alpha**	Gonal F	
				Bemfola
				Ovaleap
rFSH	G03GA06	**Follitropin beta**	Puregon	
rLH	G03GA07	**Lutropin alpha**	Luveris	
rHCG	G03GA08	**Choriogonatropin alpha**	Ovitrelle	
rFSH	G03GA09	**Corifollitropin alpha**	Elonva	
rFSH	G03GA10	**Follitropin delta**	Rekovelle	
rFSH + rLH	G03GA30	**Associations between follitropin alpha + lutropin**	Pergoveris	

**Table 4:** Comorbidities are identified from HIS through diagnosis and intervention codes ICD-9-CM in the 24 months before each cycle and/or during pregnancy

Comorbidities	ICD-9-CM definition
*Alcohol abuse*	291.xx, 303.xx, 305.0x
*Asthma*	493.x
*Cardiac valvular disease*	394.x–397.x, 424.x
*Chronic congestive heart failure*	428.22, 428.23, 428.32, 428.33, 428.42, 428.43
*Chronic ischemic heart disease*	412.x–414.x
*Chronic renal disease*	581.x–583.x, 585.x, 587.x, 588.x, 646.2x
*Congenital heart disease*	745.0x–747.4x, 648.5x
*Cystic fibrosis*	277.0x
*Drug abuse*	304.x, 305.2x–305.9x, 648.3x
*Endometriosis*	617.X
*Female infertility*	628.X
*Gestational diabetes mellitus*	648.8x (without preexisting diabetes)
*Gestational hypertension*	642.3x (without preeclampsia/eclampsia or preexisting hypertension)
*Human immunodeficiency virus*	042.x, V08.x
*Hypertension*	401.x–405.x, 642.0x–642.2x, 642.7x
*Mild preeclampsia or unspecified preeclampsia*	642.4x, 642.7x (without severe preeclampsia/eclampsia)
*Multiple gestation*	V27.2–V27.8, 651.x
*Obesity*	278.0x, 649.1x,V85.3, V85.4
*Placenta previa*	641.0x, 641.1x
*Preexisting diabetes mellitus*	250.x, 648.0x
*Previous caesarean delivery*	654.2x
*Pulmonary hypertension*	416.0x, 416.8x, 416.9x
*Severe preeclampsia/eclampsia*	642.5x, 642.6x
*Sickle cell disease*	282.4x, 282.6x
*Systemic lupus erythematosus*	710.0x
*Tobacco use*	305.1.x, 649.0x
*Tumours*	140.0–208.9, V10
*Uterine polyp*	612.0X

**Table 5:** Pregnancy interrupted and outcomes

Outcomes	Definition
*Pregnancy interrupted*	ICD-9-CM diagnosis 632–639, 761.8, 136.9, 656.4 or procedure 6859, 6901, 6902, 6909, 6951, 6952, 6993, 7491, 750, 9649, 6909, 6298
*Ovarian hyperstimulation syndrome*	ICD-9-CM 2561.x
*SGA*	Whether the infant with weight, length, and/or head circumference at birth below the 10th percentile of the reference distribution for gestational age and sex*
*LBW*	A birth weight of an infant of 2,499 g or less
*PRETERM*	A baby born before 37 weeks of gestation

^*^[14]
